# Evaluating ambroxol as an agent to boost lysosomal function and reduce beta amyloid in 3xTg mice

**DOI:** 10.1002/alz.093495

**Published:** 2025-01-03

**Authors:** Adrianna R Tsang, Lynn Wang, Sairam Gajavelli, Stephen H Pasternak

**Affiliations:** ^1^ University of Western Ontario, London, ON Canada

## Abstract

**Background:**

Ambroxol is an expectorant under study as a treatment for synucleinopathies, such as Parkinson’s disease. It is a pharmacological chaperone of the lysosomal enzyme β‐glucocerebrosidase (GCase), increasing this enzyme and subsequently reducing accumulation of alpha‐synuclein. Although the mechanism of enhanced clearance is not fully understood, ambroxol stimulates lysosomal function through activation of transcription factor EB (TFEB), which drives hundreds of lysosomal genes. This project aims to investigate the usefulness of ambroxol as a disease‐modifying treatment for Alzheimer’s disease (AD). We hypothesize that ambroxol will enhance expression of lysosomal proteins and increase clearance of beta‐amyloid (Aβ) in the 3xTg mouse model of AD.

**Methods:**

Wild‐type (B6;129) or 3xTg mice (APP/PS1/MAPT) mice were used. A ‘prevention’ group was treated at 6 months of age for 2 months, during initial Aβ deposition. A second ‘treatment group’ was fed ambroxol from 8 to 10 months of age, after Aβ deposits had developed. Mice received either 1) normal mouse chow, 2) low dose (1200 mg/kg) chow, or 3) or high dose (2400 mg/kg) chow. Brains were collected for immunohistochemistry, western blotting and ELISAs.

**Results:**

By immunostaining, mean expression of ambroxol treated animals demonstrated increased GCase and LAMP1 staining, as predicted. This was accompanied by a significant dose dependant reduction of Aβ in aged 3xTg mice.

**Conclusions:**

Our findings demonstrate that ambroxol is able to reduce intracellular accumulation of Aβ by upregulating lysosomal activity, and therefore may be a promising treatment for AD.

